# Exogenous 5-azaCitidine accelerates flowering and external GA_3_ increases ornamental value in Iranian *Anemone* accessions

**DOI:** 10.1038/s41598-021-86940-6

**Published:** 2021-04-05

**Authors:** Vahideh Yari, Zeynab Roein, Atefeh Sabouri

**Affiliations:** 1 Department of Horticultural Sciences, Faculty of Agriculture, Ilam University, Ilam, Iran; 2 Department of Agronomy and Plant Breeding, Faculty of Agricultural Sciences, University of Guilan, Rasht, Iran

**Keywords:** Plant sciences, Plant physiology

## Abstract

The *Anemone* genus is a tuberous geophyte which undergoes a dormancy period during unfavorable environmental conditions for growth. Five species of the *Anemone* genus naturally grow in several regions of Iran. The diverse uses of *Anemone* in gardens for landscaping, cut flowers, and potted plants indicate its high ornamental potential. Its dormancy and flowering are influenced by various factors. The present paper was conducted to explore the flowering behavior of *Anemone* accessions in response to different pre-treatments. For this purpose, tubers of 18 *Anemone* accessions (*A. coronaria* and *A. biflora*) were collected from natural regions of six provinces in Iran. These tubers were subjected to different conditions of non-chilling (20 °C, 90 days), chilling (4 °C, 90 days), GA_3_ (150 mgL^-1^; 24 h), and 5-azaCitidine (5-azaC; 40 µM; 24 h) prior to the cultivation. Most of the accessions were able to enter the flowering stage without chilling. The shortest period for the sprouting of tubers (16.89 ± 7.83 days) belonged to 5-azaC pre-treatment. In addition, this treatment accelerated the flowering time (about 30 days earlier) and diameter of the stem, bud, and flower. Morphological characteristics, such as stem height, number of leaves, bud, and petal and the longevity of flowers on the plant were significantly affected by GA_3_ pre-treatment. Our results indicated a positive correlation between flower length, stem height, and stem diameter with flower longevity under different pre-treatment conditions. The present study demonstrated that accessions Anm3, Anm12, and Anm18 had ornamental values higher than the population mean across four conditions.

## Introduction

The genus *Anemone* belonging to the Ranunculaceae family comprises several species of perennial ornamental plants. This geophyte is mainly distributed in the Mediterranean region^1,2^. *Anemone coronaria* is a major tuberous species of this genus with a wide variety of colors (red, white, pink, blue, and violet) and great horticultural importance. This ornamental plant is used for a variety of purposes as cut flowers, potted plants, and garden plants^3,4^. In the Mediterranean climate, the typical life cycle of *Anemone* starts in the autumn, followed by active growth and flowering in winter and early spring. A period of dry and warm summer induces tuber dormancy^4,5^. With colorful flowers, this attractive plant belongs to the group of highly ornamental geophytes distributed as wild flowers over a wide range of areas in Iran^6^. Taxonomic studies have found several wild *Anemone* species in Iran, including *A. biflora*, *A. caucasica*, *A. coronaria*, *A. petiolulsa*^1^, and *A. narcissiflora*^7^. These plants may grow in different areas with arid and semi-arid climates with mild winters and hot summers to cold regions with very cold and longer winters. Their growth and flowering period in different habitats of Iran ranges from February to mid-June. The flowering period ends before the high environmental temperature during late spring. As the long days and warm season commence, the plants enter the dormancy stage. The species of *Anemone* are widely grown in very shallow to mid-deep soils with a light texture^8^.

Accelerating the flowering process and decreasing the vegetative period while maintaining the quality of flowers in geophyte plants are among the main needs of growers in greenhouse conditions. Forcing is a set of techniques used by growers to regulate and modify plant development^9,10^; this process requires knowledge about the dormancy release mechanism of the plants. Exposure to low temperature is one of the most important environmental signals causing biochemical and physiological changes, inducing responses, such as dormancy release, stem elongation, and flowering in spring flower geophytes^8^. Fulfilling the chilling requirement of geophytes for off-season blooming is a time-consuming process. Therefore, introducing a suitable alternative to low temperature could accelerate the floral transition and reduce the cost of long-term cold treatment and the energy consumption in the greenhouse during year-round commercial production^11^.

Gibberellins (GAs) are a group of phytohormones that regulate various developmental stages, for instance, germination, root elongation, plant growth facilitation, and transition to the reproductive phase of plants^10,12–14^. GA_3_ is one of the best compounds for increasing the vegetative parameters^15^, accelerating flower bud development, regulating branching, and enhancing the quality of flowers^16,17^. Additionally, GA_3_ is able to function at low temperatures and promote dormancy release^18,19^. Several reports have revealed that GA_3_ could promote flowering in *Epipremnum aureum*^20^, and influence flowering in *Kalanchoe*^21^, *Paeonia*^9,10^, and *Chrysanthemum*^22^. In another study, Ramzan et al.^23^ showed that the treatment of tulip bulbs with GA_3_ (150 mg L^-1^) reduced the production period in greenhouse.

Furthermore, it has been reported that the vernalization process is accompanied by DNA demethylation^24^. DNA methylation refers to the enzymatic addition of a methyl group of S-adenosyl methionine (SAM) by DNA methyltransferases onto the C5 position of cytosine, which increases 5-methyl cytosine (5 mC) in genomic DNA^25,26^. The percentage of DNA methylation changes during the flower development^27^. It is possible to reduce DNA methylation via specific inhibitors that limit DNA methyltransferases activity ^28,29^. Azacitidine (5-azaC) is a known cytosine analogue that substitutes cytosine and is randomly inserted into newly synthesized DNA strands^30^. Following the application of 5-azaC, phenotypic variation^31^, dwarfism, and early flowering^32^ were observed in *Jatropha curcas* and *Pharbitis nil* plants. Moreover, reports have implied that changes in DNA methylation influence the flower shape and stem color variation in *Chrysanthemum lavandulifolium*^33^. Exposure of grape berries^34^ and *Spinacia oleracea*^35^ to 5-azaC results in the acceleration of ripening and flowering time whereas exogenous application of 5-azaC on *Paeonia suffruticosa* improves bud sprouting^29^.

Wild ornamental plant types are negatively affected by land-cover changes, invasive species, and environmental factors. Therefore, conservation and maintenance of genetic resources of ornamental plants is essential to breeding and future development^36^. To conserve the biodiversity of endangered plants outside natural habitats, ex situ conservation^37^ has been proposed. Furthermore, sustainable use of rare and endemic species is one of the best alternatives to preservation^36^. Wild species are important genetic resources since they can carry genes responsible for further improvement in various ornamental traits and tolerance to various biotic and abiotic stresses^36^. In addition, the identification of promising native plant genetic resources for domestication purposes requires long-term plans for collection, conservation, and characterization^38^. Accordingly, genetic diversity is an important component of conservation biodiversity^39^. Evaluation and characterization of genetic diversities existing in the wild *Anemone* germplasm is an important step toward selecting efficient germplasm management systems. Despite the widespread distribution of *Anemone* in different regions of Iran^1^, the diversity and physiology of flowering and dormancy parameters have not been studied yet. Investigation and detection of variations among different accessions is a critical step before starting a breeding project^40^. Thus, enhancing the diversity of *Anemone* through conventional breeding requires the assessment of morpho-phenological diversity among *Anemone* accessions so that they could be exploited for the required traits. Due to the scarcity of knowledge concerning the growth and development of *Anemone* accessions, it is important to assess the effect of hormonal and chilling pre-treatments on their flowering and dormancy behavior with high statistical estimation. Therefore, obtaining information concerning the tuber dormancy mechanism and sprouting behavior of *Anemone* may lead to a better design of an efficient forcing management system. In addition, it could be hypothesized that optimal flowering time is obtained using different pre-treatments (5-azaC and GA_3_). The main objective of this research was to identify superior accessions within germplasm collection under pre-treatment conditions. Additionally, the present study aimed to answer the following questions: (i) Is chilling required for flowering in different *Anemone* accessions? (ii) Can pre-treatment with GA_3_ and 5-azaC replace chilling? (iii) Does 5-azaC affect the morpho-phenolological characteristics of the *Anemone* plants? (iv) Which attributes are suitable for selecting superior accessions?

## Results

### Analysis of variance

The effects of the accessions, pre-treatments, and accessions × pre-treatment interaction were studied by conducting a factorial experiment as CRD design. The analysis of variance (ANOVA) revealed significant differences (*P* < 0.01) among the accessions, pre-treatments, and accessions × pre-treatment interaction regarding almost all the evaluated characteristics (Table 1). According to this analysis, there was a significant difference among *Anemone* accessions in response to different pre-treatment conditions for breaking the tuber dormancy and flowering. In other words, different reactions were observed for the accessions in response to various pre-treatments. Therefore, to determine the differences between the accessions in more detail, the means and Z-scores were separately compared in each pre-treatment.

**Table 1:** Analysis of variance (ANOVA) for measured morpho-phenological traits of 18 studied of *Anemone* accessions under different pre-treatment conditions.

Source of variation	df	Mean square																	
		Sprouting time	Bud stage	Bud stage in color	Flower anthesis	Flower longevity	Flower bud number	Bud length	Bud diameter	Leaf number	Leaf width	Leaf length	Petal number	Flower length	Flower diameter	Stem height	Stem diameter	Root length	Tuber fresh weight
Accession	17	1119**	1996 **	26.50**	33.40**	22.27**	0.67**	50.90**	21.09**	4.08**	307.20**	119.50**	7.23**	129.60**	473.70**	11,412**	1.65**	3087**	9.23**
Pre-treatment	3	1023**	13,909**	53.70**	22.80**	36.87**	0.45*	52.90**	30.40**	3.02*	43.60 ns	8.520 ns	10.97**	252.10**	698.60**	11,453**	2.39**	1573 ns	0.18 ns
Accession × Pre-treatment	51	342.80**	2442 **	34.50**	19.70**	13.38**	0.34**	38.00**	17.01**	3.06**	351.40**	201.40**	5.75**	104.90**	304.20**	5058 **	1.12**	6850**	7.24**

ns (Non-significant), *(Significant at 5% probability level) and **(Significant at 1% probability level). Pre-treatment conditions included non-chilling, chilling, 5-azaC and GA_3_.

### Evaluation of different pre-treatments on phenological traits in *Anemone* accessions

**Figure 1:**
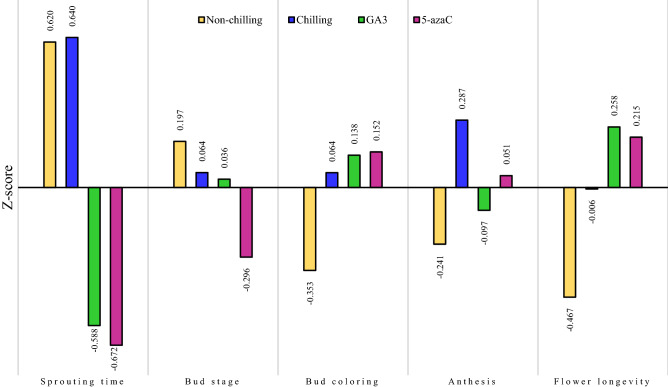
Distribution of the values of the Z-scores for phenological traits under different pre-treatment conditions in 18 studied accessions of *Anemone*. Z-score was calculated as the difference between the mean of the trait (for each pre-treatment) and the total mean, divided by the total standard deviation (SD). The highest negative Z-scores are associated with the relative lowest value of the variable among the conditions. Since the decrease in the value of phenological traits (except for flower longevity on plant) is a desirable feature, the pre-treatments with more negative Z-scores were considered as more favorable for increasing the rate of plant growth.

The effects of four pre-treatment conditions (non-chilling, chilling, 5-azaC, and GA_3_) on the phenological traits of 18 *Anemone* accessions were investigated (Table 2). As seen, the fastest sprouting time belonged to 5-azaC and GA_3_ pre-treatment. The shortest period to the sprouting of *Anemone* tubers (16.89 ± 7.83 days) was recorded for 5-azaC pre-treatment, which occurred approximately 18 days earlier than that in the non-chilling condition (no pre-treatment was applied to the tubers). Moreover, 24 h-immersion of the tubers in GA_3_ solution accelerated sprouting to 18 days. On the other hand, the longest sprouting time was recorded for chilling and non-chilling pre-treatments lasting 34.98 ± 11.82 and 34.71 ± 15.39 days, respectively. Concerning the Z-score of the pre-treatments (Fig. 1), it was found that 5-azaC with a more negative Z-score (-0.672) was more effective on reducing the sprouting time than the flower bud formation time (Z-score = -0.29). It should be noted that the decrease or negative changes in the value of phenological traits (except for flower longevity on plant) are desirable features for horticultural researchers. Thus, the pre-treatments with further negative Z-scores were considered to be more favorable for increasing the rate of plant growth. A Z-score indicates the number of standard deviations by which the value of the mean of the treatment group is above or below the total mean value. The results also revealed that flower bud formation in 5-azaC occurred earlier than that in other pre-treatments (Table 2). The tubers treated with 5-azaC entered the budding stage about two weeks earlier than that in the non-chilling conditions. In contrast, flower bud color was detectable earlier in the no pre-treatment tubers (with maximum negative Z-score, -0.35); accordingly, less than 10 days following bud formation, bud color became visible. However, based on these findings, GA_3_ and 5-azaC pre-treatments required more than 11 days to show bud coloring (Table 2, Fig. 1). Additionally, the full opening of the flower in the non-chilling pre-treatment was faster (Z-score = -0.24) than that in other pre-treatments. According to the results, the longest period for flower opening after bud coloring belonged to the chilled tubers, which lasted about 8.3 days. Moreover, with 5-azaC, it took 7.6 days for the flower to fully open. On the contrary to the non-chilling pre-treatment (Z-score = -0.47), the highest longevity of flowers on the plant was recorded for GA_3_ (Z-score = 0.26) and 5-azaC (Z-score = 0.21), respectively. Overall, the comparisons of the effects of pre-treatments among all the phenological traits showed that 5-azaC accelerated the flowering time of *Anemone* accessions the most.

**Table 2:** Mean values ± SD for measured phenological traits under different pre-treatment conditions in 18 studied accessions of *Anemone*.

Traits	Pre-treatment			
	Non-chilling	Chilling	GA3(150 mg L^−1^)	5-azaC (40 µM)
Sprouting time (day)	34.71 ± 15.39	34.98 ± 11.82	18.05 ± 5.96	16.89 ± 7.83
Bud stage (day)	79.62 ± 45.56	75.81 ± 21.72	75.02 ± 19.35	65.54 ± 19.12
Bud stage in color (day)	9.58 ± 4.66	10.98 ± 3.57	11.23 ± 2.60	11.28 ± 1.92
Flower anthesis (day)	6.81 ± 4.05	8.30 ± 2.67	7.21 ± 2.06	7.63 ± 2.03
Flower longevity (day)	5.62 ± 3.07	6.70 ± 2.29	7.32 ± 1.87	7.22 ± 1.71

### Evaluation of different pre-treatments on morphological characteristics in *Anemone* accessions

**Figure 2:**
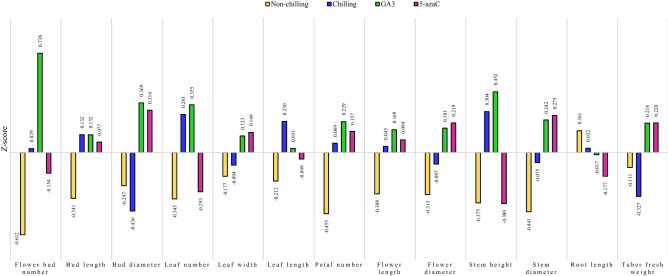
Distribution of the values of the Z-scores for morphological traits under different pre-treatment conditions in 18 studied accessions of *Anemone*. Z-score was calculated as the difference between the mean of the trait (for each pre-treatment) and the total mean, divided by the total standard deviation (SD). The highest positive Z-scores are associated with the relative highest value of the variable among the conditions. Since increase in the value of morphological traits is a desirable feature, pre-treatments with further positive Z-scores were considered as more favorable for increasing the rate of plant growth.

As represented in Fig. 2, GA_3_ had a more positive Z-score than that in other pre-treatments associated with most morphological traits, such as the number of flower buds, the number of petals, stem height, flower length, and leaf number. This means that GA_3_ was effective on increasing the plant size. On the contrary, 5-azaC, with a negative Z-score (Fig. 2), reduced the flower bud number, leaf number, stem height, and root length. However, it had a positive Z-score for flower bud diameter, flower diameter, stem diameter, and tuber fresh weight compared to other pre-treatments. These results were obtained while the tubers with no pre-treatments before the cultivation had the most negative Z-scores for the assessed traits (Fig. 2). Therefore, the lack of chilling in the tubers resulted in a decrease in the size and dimensions of plant in comparison to other pre-treatments. In contrast, root length was longer in non-chilling conditions than that in other treatments. Overall, for morphological traits, GA_3_ pre-treatment with the maximum positive Z-score was more effective on increasing the plant size as compared with other pre-treatments (Table 3; Fig. 2).

**Table 3:** Mean values ± SD for morphological traits under different pre-treatment conditions in 18 studied accessions of *Anemone.*

Traits	Pre-treatment			
	Non-chilling	Chilling	GA3(150 mg L^−1^)	5-azaC (40 µM)
Flower bud number	0.81 ± 0.42	1.07 ± 0.39	1.36 ± 0.43	1.00 ± 0.11
Bud length (mm)	8.49 ± 4.79	10.30 ± 3.44	10.30 ± 3.48	10.09 ± 3.42
Bud diameter (mm)	5.61 ± 3.34	5.12 ± 1.51	7.18 ± 2.43	7.04 ± 2.13
Leaf number	8.55 ± 4.89	10.74 ± 3.08	10.99 ± 2.70	8.73 ± 2.21
Leaf width (mm)	22.16 ± 11.27	23.03 ± 10.35	25.30 ± 10.32	25.57 ± 10.39
Leaf length (mm)	22.33 ± 10.16	25.72 ± 7.74	24.19 ± 6.08	23.58 ± 6.24
Petal number	4.19 ± 2.13	4.96 ± 1.42	5.19 ± 1.10	5.09 ± 0.72
Flower length (mm)	14.78 ± 8.55	17.03 ± 5.52	17.82 ± 5.92	17.34 ± 5.06
Flower diameter (mm)	26.09 ± 15.53	28.60 ± 9.40	31.57 ± 9.58	32.00 ± 8.32
Stem height (mm)	103.40 ± 59.44	136.02 ± 45.88	143.12 ± 44.63	103.14 ± 24.45
Stem diameter (mm)	1.69 ± 0.94	1.93 ± 0.55	2.14 ± 0.53	2.17 ± 0.46
Root length (mm)	118.02 ± 50.63	112.28 ± 48.58	110.12 ± 45.20	103.01 ± 33.98
Tuber fresh weight (g)	3.55 ± 1.77	3.21 ± 1.07	4.08 ± 1.64	4.08 ± 1.77

### Clustering and identifying superior accessions under non-chilling condition

In an attempt to better identify the response of each accession to different pre-treatments, the present set of accessions were separately grouped and analyzed in each pre-treatment condition. Four clusters were identified in the non-chilling condition (Supplementary Fig. 1). The maximum number of accessions (eight accessions) were assigned to cluster I that had different sources (Table 4). Cluster I consisted of Anm3, Anm9, Anm10, Anm11, Anm12, Anm13, Anm15, and Anm18, whose mean values in terms of most traits were higher than the total mean (Table 4). In other words, in the absence of chilling, these accessions were identified to be superior. This cluster represented the accessions with a lower Z-score for sprouting time than the accessions in other clusters. Z-score comparison for different traits showed that the members of this group had a relative advantage over other accessions with larger aerial parts and early sprouting. Cluster II included accessions Anm1, Anm4, Anm7, Anm14, and Anm17; the most prominent feature of this group was the long time of sprouting. Cluster III, with two accessions of Anm6 and Anm8, had a negative deviation from the total mean for most of the traits. This means that the members of this group had low-quality traits compared to other accessions under non-chilling conditions. For note, the accessions in this group had relatively long roots. In contrast, the values in Table 4 depicts that cluster IV also contained two accessions (Anm5 and Anm16) that failed to enter the flowering phase despite early sprouting. Negative Z-score for root length and tuber fresh weight indicated that the underground organs in these accessions were limited under non-chilling conditions and their growth was slow (Table 4). Interestingly, accession Anm2 was unable to sprout under these conditions; thus, it did not fit into any of the clusters.

**Table 4:** Composition of the groups and the distribution of the *Anemone* accessions in each group based on cluster analysis under non-chilling pre-treatment.

Cluster	Accession code		Sprouting time (day)	Bud stage (day)	Bud stage in color (day)	Flower anthesis (day)	Flower longevity (day)	Flower bud number	Bud length (mm)	Bud diameter (mm)	Leaf number
I	Anm3, Anm9, Anm10, Anm11, Anm12, Anm13, Anm15, Anm18	Group mean	31.71	102.25	11.69	9.83	7.67	1.00	11.70	7.50	11.70
		Z-score*	− 0.38	0.42	0.37	0.69	0.61	0.37	0.61	0.50	0.61
II	Anm1, Anm4, Anm7, Anm14, Anm17	Group mean	50.77	101.10	11.60	7.30	6.70	1.06	9.56	7.06	9.83
		Z-score	1.07	0.39	0.35	0.02	0.26	0.54	0.13	0.35	0.17
III	Anm6, Anm8	Group mean	34.67	54.83	10.50	3.67	3.17	0.67	5.68	2.77	5.00
		Z-score	− 0.16	− 0.7	0.08	− 0.9	− 0.98	− 0.52	− 0.74	− 1.01	− 0.89
IV	Anm5, Anm16	Group mean	24.00	0.00	0.00	0.00	0.00	0.00	0.00	0.00	0.00
		Z-score	− 0.97	− 1.99	− 2.45	− 1.9	− 2.11	− 2.31	− 2.02	− 1.89	− 1.99
Total mean			36.75	84.30	10.15	7.21	5.95	0.86	8.90	5.94	9.05

Cluster	Accession code		Leaf width (mm)	Leaf length (mm)	Petal number	Flower length (mm)	Flower diameter (mm)	Stem height (mm)	Stem diameter (mm)	Root length (mm)	Tuber fresh weight (g)
I	Anm3, Anm9, Anm10, Anm11, Anm12, Anm13, Anm15, Anm18	Group mean	28.19	26.18	5.35	18.54	36.06	142.52	2.16	102.41	3.57
		Z-score*	0.47	0.29	0.47	0.58	0.36	0.60	0.43	− 0.53	− 0.12
II	Anm1, Anm4, Anm7, Anm14, Anm17	Group mean	20.84	25.27	5.40	20.25	32.11	124.64	2.29	160.99	4.88
		Z-score	− 0.26	0.18	0.50	0.58	0.31	0.27	0.58	0.85	0.71
III	Anm6, Anm8	Group mean	21.75	24.66	2.83	8.21	10.27	48.88	0.84	141.85	3.28
		Z-score	− 0.17	0.12	− 0.83	− 0.93	− 1.19	− 1.10	− 1.10	0.40	− 0.30
IV	Anm5, Anm16	Group mean	12.79	8.34	0.00	0.00	0.00	0.00	0.00	108.23	2.19
		Z-score	− 1.05	− 1.74	− 2.31	− 1.96	− 1.90	− 1.98	− 2.08	− 0.39	− 0.98
Total mean			23.46	23.64	4.44	15.65	27.62	109.48	1.79	124.96	3.76

*: Z-score was calculated as the difference between the mean of the trait (for each group) and the total mean, divided by the total standard deviation (SD). The highest positive Z-scores are associated with the relative highest value of the variable among the groups. Since the decrease in the value of phenological traits (except for flower longevity on plant) is a desirable feature, the groups with further negative Z-scores were considered to be more superior. Conversely, since the increase in the value of morphological traits is a desirable feature, the groups with further positive Z-scores were considered to be superior.

### Clustering and identifying superior accessions under artificial chilling conditions

The dendrogram of 18 *Anemone* accessions under artificial chilling condition was constructed based on morpho-phenological parameters (Supplementary Fig. 2). In general, cold exposure (90 days at 4 °C) during the storage of the tubers prior to the planting had an adverse effect on the growth and flowering of most accessions. Table 5 represents the results associated with the cluster analysis of the accessions under chilling conditions, in which the accessions are divided into four clusters. Among them, cluster II with six accessions (Anm3, Anm5, Anm12, Anm14, Anm17, and Anm18) had a positive deviation from the total mean related to all the morphological traits except for flower bud size and leaf number (Table 5). Furthermore, this cluster had a negative Z-score for sprouting time under chilling conditions. This implied the superiority of this cluster in improving the morphological traits and reducing the time for sprouting. On the contrary, the first group (cluster I with eight accessions) had values lower than the total mean for most morphological and phenological traits. The third group (cluster III with Anm1 and Anm16) was superior to the other groups regarding phenological traits and the short flowering period. The fourth group (cluster IV with one accession; Anm7) had larger underground parts and flower bud, but it had a longer sprouting time compared with other accessions (Table 5). This accession, well-separated from other groups under chilling conditions, belonged to *A. biflora* species. Similar to non-chilling conditions, sprouting for accession Anm2 did not occur under artificial chilling conditions while accessions Anm5 and Anm16 entered the flowering stage once they received cold treatment.

**Table 5:** Composition of the groups and the distribution of the *Anemone* accessions in each group based on cluster analysis under chilling pre-treatment.

Cluster	Accession code		Sprouting time (day)	Bud stage (day)	Bud stage in color (day)	Flower anthesis (day)	Flower longevity (day)	Flower bud number	Bud length (mm)	Bud length (mm)	Leaf number
I	Anm4, Anm6, Anm8, Anm9, Anm10, Anm11, Anm13, Anm15,	Group mean	38.08	84.12	12.04	9.00	7.33	1.08	12.36	12.36	12.33
		Z-score*	0.13	0.35	0.18	0.12	0.15	− 0.19	0.62	0.62	0.61
II	Anm3, Anm5, Anm12, Anm14, Anm17, Anm18	Group mean	34.22	81.50	12.11	9.61	7.28	1.33	11.09	11.09	11.17
		Z-score	− 0.34	0.11	0.21	0.48	0.11	0.68	0.08	0.08	− 0.13
III	Anm1, Anm16	Group mean	35.83	62.83	8.33	5.67	6.50	0.83	7.15	7.15	9.00
		Z-score	− 0.15	− 1.59	− 1.40	− 1.80	− 0.37	− 1.05	− 1.60	− 1.60	− 1.52
IV	Anm7	Group mean	48.00	77.00	12.00	8.33	5.33	1.00	5.71	5.71	9.67
		Z-score	1.34	− 0.30	0.16	− 0.26	− 1.10	− 0.47	− 2.21	− 2.21	− 1.09
Total mean			37.04	80.27	11.63	8.78	7.10	1.14	10.90	10.90	11.37

Cluster	Accession code		Leaf width (mm)	Leaf length (mm)	Petal number	Flower length (mm)	Flower diameter (mm)	Stem height (mm)	Stem diameter (mm)	Root length (mm)	Tuber fresh weight (g)
I	Anm4, Anm6, Anm8, Anm9, Anm10, Anm11, Anm13, Anm15,	Group mean	22.77	27.00	5.46	19.55	29.44	143.48	1.98	99.48	3.14
		Z-score*	− 0.18	− 0.05	0.29	0.42	− 0.13	− 0.02	− 0.26	− 0.47	− 0.34
II	Anm3, Anm5, Anm12, Anm14, Anm17, Anm18	Group mean	30.65	30.27	5.55	18.41	35.24	163.93	2.31	139.79	3.73
		Z-score	0.71	0.68	0.41	0.10	0.79	0.63	0.96	0.51	0.46
III	Anm1, Anm16	Group mean	19.36	25.36	3.50	13.36	23.11	113.22	1.62	120.70	3.07
		Z-score	− 0.57	− 0.42	− 2.43	− 1.29	− 1.14	− 0.97	− 1.59	0.04	− 0.45
IV	Anm7	Group mean	9.76	14.58	5.33	12.96	21.67	90.45	1.90	145.11	4.04
		Z-score	− 1.65	− 2.83	0.11	− 1.40	− 1.37	− 1.68	− 0.54	0.64	0.88
Total mean			24.39	27.23	5.25	18.03	30.29	144.02	2.05	118.88	3.39

*: Z-score was calculated as the difference between the mean of the trait (for each group) and the total mean, divided by the total standard deviation (SD). The highest positive Z-scores are associated with the relative highest value of the variable among the groups. Since the decrease in the value of phenological traits (except for flower longevity on plant) is a desirable feature, the groups with further negative Z-scores were considered to be more superior. Conversely, since the increase in the value of morphological traits is a desirable feature, the groups with further positive Z-scores were considered to be superior.

### Clustering and identifying superior accessions under GA_3_ immersion

Based on the cluster analysis of accessions under a 24-h immersion with GA_3_ prior to the planting, the clear distinction among accessions occurred when they were grouped into three major clusters (Supplementary Fig. 3). The 11 accessions (including Anm1, Anm3, Anm5, Anm8, Anm9, Anm10, Anm12, Anm13, Anm14, Anm15 and Anm18) characterized by early sprouting ability and positive Z-score for the aerial parts (Table 6) were assigned to the first cluster (I). These characteristics indicate the superiority of the accessions in this group in terms of ornamental features. Moreover, the members of the same group had negative Z-scores for the tuber weight and root length, implying the weakness of the accessions in the underground parts. The prominent indices of the second group (cluster II) consisted of five accessions (Anm2, Anm4, Anm7, Anm11 and Anm17); these accessions had strong underground parts, such as long root and weighty tuber. This group did not score well for sprouting time and most of the morphological traits associated with flower section. In contrast, accessions Anm6 and Anm16 with negative Z-scores for most of the traits (except for root length) belonged to the third group (cluster III). Evidently, these accessions differed from the others, and this cluster was the weakest group under tuber immersion in GA_3_ (Table 6). An interesting point in this section was the effect of GA_3_ on accession Anm2, which induced sprouting and flowering in this accession.

**Figure 3:**
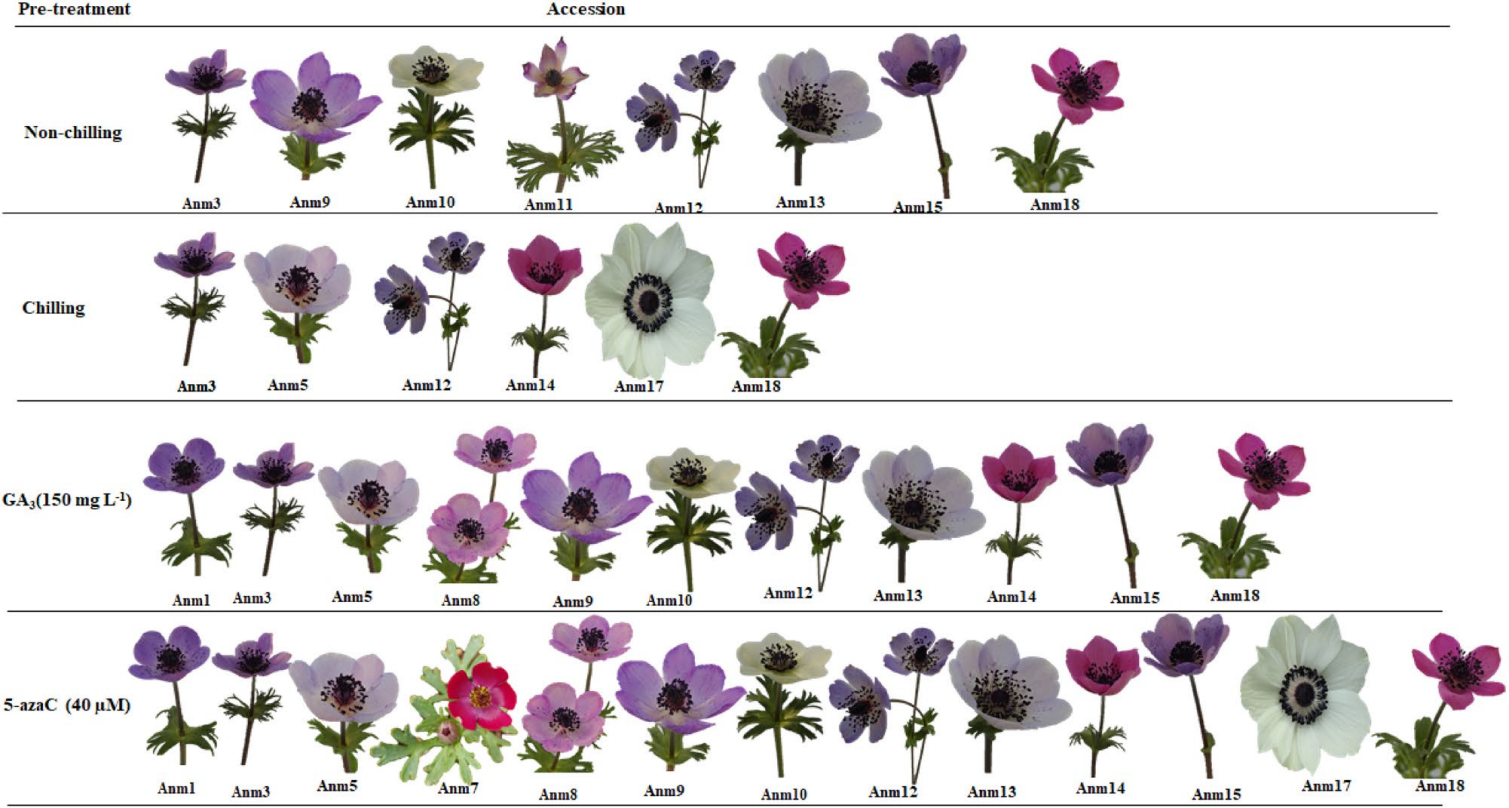
Comparison of superior *Anemone* accessions under different pre-treatment conditions (non-chilling, chilling, 5-azaC and GA_3_). The flower of each genotype was used as a symbol. By 5-azaC and chilling pre-treatments, the superior group comprised respectively the highest (13) and lowest (6) number of accessions which presented relatively the most desirable value of the traits among the groups. (Images were prepared in Microsoft PowerPoint, 2016).

**Table 6:** Composition of the groups and the distribution of the *Anemone* accessions in each group based on cluster analysis under GA_3_ pre-treatment.

Cluster	Accession code		Sprouting time (day)	Bud stage (day)	Bud stage in color (day)	Flower anthesis (day)	Flower longevity (day)	Flower bud number	Bud length (mm)	Bud diameter (mm)	Leaf number
I	Anm1, Anm3, Anm5, Anm8, Anm9, Anm10, Anm12, Anm13, Anm14, Anm15, Anm18	Group mean	15.68	75.15	12.11	7.56	7.77	1.44	11.98	7.85	12.56
		Z-score*	− 0.39	0.007	0.33	0.17	0.24	0.18	0.48	0.27	0.58
II	Anm2, Anm4, Anm7, Anm11, Anm17	Group mean	22.13	82.93	10.93	7.40	7.80	1.40	9.14	7.49	9.13
		Z-score	0.68	0.41	− 0.11	0.09	0.25	− 0.09	0.33	0.13	− 0.68
III	Anm6, Anm16	Group mean	20.83	54.50	7.16	4.83	3.67	0.83	3.93	2.75	7.00
		Z-score	0.46	− 1.06	− 1.56	− 1.15	− 1.95	− 1.23	− 1.82	− 1.82	− 1.47
Total mean			18.05	75.02	11.23	7.21	7.32	1.36	10.29	7.18	10.99

Cluster	Accession code		Leaf width (mm)	Leaf length (mm)	Petal number	Flower length (mm)	Flower diameter (mm)	Stem height (mm)	Stem diameter (mm)	Root length (mm)	Tuber fresh weight (g)
I	Anm1, Anm3, Anm5, Anm8, Anm9, Anm10, Anm12, Anm13, Anm14, Anm15, Anm18	Group mean	27.63	26.77	5.53	20.01	34.67	164.97	2.37	96.79	3.94
		Z-score*	0.22	0.42	0.30	0.37	0.32	0.49	0.43	− 0.29	− 0.08
II	Anm2, Anm4, Anm7, Anm11, Anm17	Group mean	22.37	20.72	5.53	17.41	32.30	130.93	2.08	126.34	5.16
		Z-score	− 0.28	− 0.57	0.31	− 0.07	0.07	− 0.27	− 0.10	0.36	0.65
III	Anm6, Anm16	Group mean	19.79	18.66	2.50	6.77	12.68	53.41	1.02	142.86	2.12
		Z-score	− 0.53	− 0.90	− 2.44	− 1.86	− 1.97	− 2.01	− 2.10	0.72	− 1.19
Total mean			25.29	24.19	5.19	17.82	31.57	143.12	2.14	110.12	4.08

*: Z-score was calculated as the difference between the mean of the trait (for each group) and the total mean, divided by the total standard deviation (SD). The highest positive Z-scores are associated with the relative highest value of the variable among the groups. Since the decrease in the value of phenological traits (except for flower longevity on plant) is a desirable feature, the groups with further negative Z-scores were considered to be more superior. Conversely, since the increase in the value of morphological traits is a desirable feature, the groups with further positive Z-scores were considered to be superior.

### Clustering and identifying superior accessions under 5-azaC application

According to cluster analysis under a 24-h immersion of tuber with 5-azaC, the biggest difference among the groups was related to when the accessions were divided into four main clusters (Supplementary Fig. 4). Under these conditions, the first group (cluster I) with 13 accessions (Anm1, Anm3, Anm5, Anm7, Anm8, Anm9, Anm10, Anm12, Anm13, Anm14, Anm15, Anm17, and Anm18) was the largest one (Table 7). Based on the comparison of the Z-scores of the traits, it could be inferred that the members of this group had taller plants with larger leaves and flowers. The superior accessions were placed in cluster I and characterized by their higher ornamental value under 5-azaC application. Meanwhile, the second group (cluster II), comprising two accessions (Anm2 and Anm4), entered the flowering stage (with early sprouting) faster than that in other groups under 5-azaC application. This group had a relative superiority in terms of flower longevity on plant and tuber weight compared over the other groups. The most distinguishing feature in the third group (cluster III), with one accession (Anm11), was the large number of flower buds. Similar to the grouping of GA_3_ condition, accessions Anm6 and Anm16 were clustered together (cluster IV), which is indicative of their poor response to 5-azaC. Plant size and phenology were negatively affected by 5-azaC; therefore, the general characteristics of the two accessions were late sprouting and small plant size. On the other hand, this group had longer roots once exposed to 5-azaC (Table 7). It is noteworthy that the effect of 5-azaC was similar to that of GA_3_ on accession Anm2, where the 24-h immersion in 5-azaC caused sprouting and flowering in this accession.

**Figure 4:**
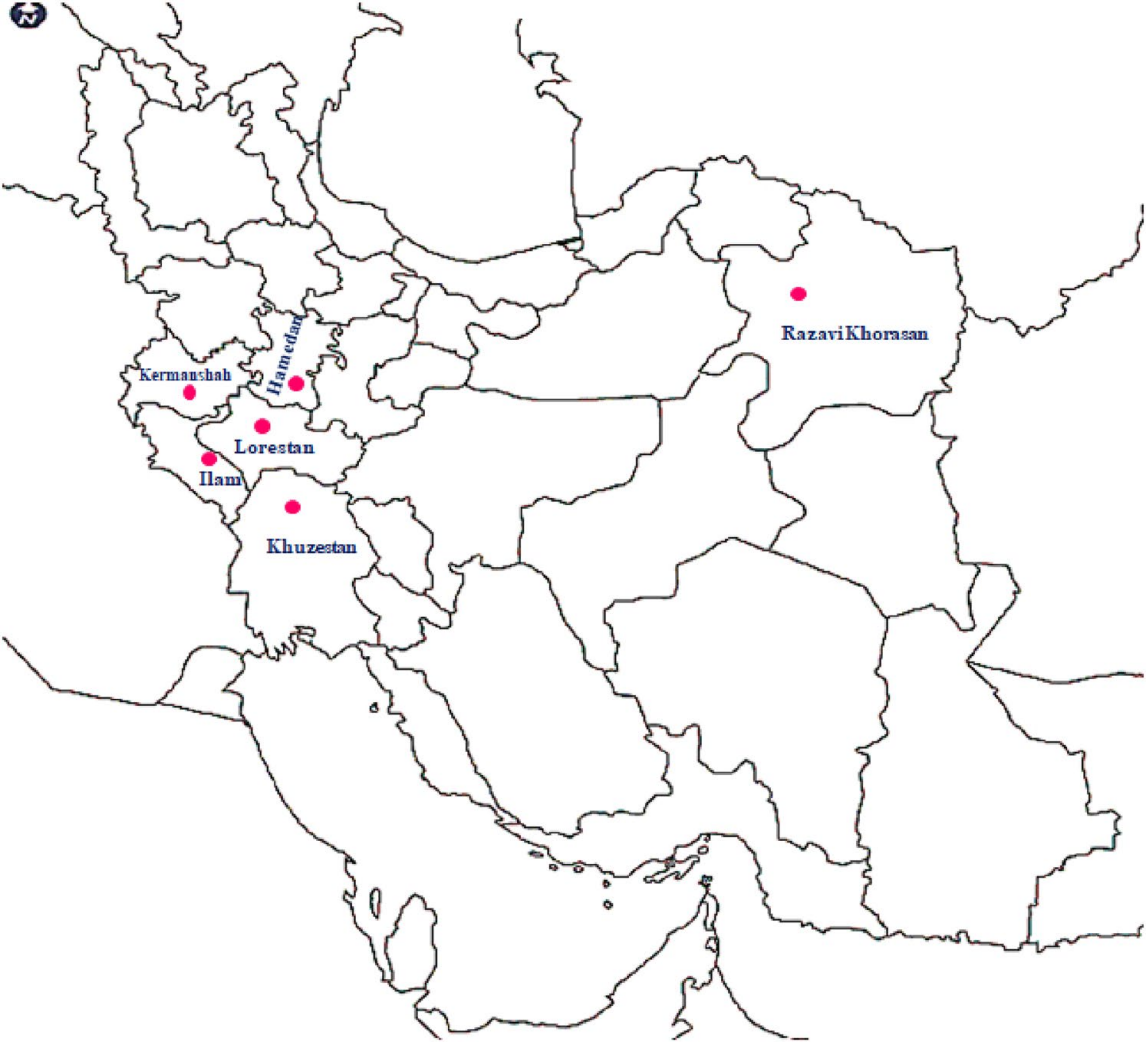
The provinces where the *Anemone* accessions were collected and their locations in Iran. The total area of the country: 1.648 million km^2^.

**Table 7:** Composition of the groups and the distribution of the *Anemone* accessions in each group based on cluster analysis under 5-azaC pre-treatment.

Cluster	Accession code		Sprouting time (day)	Bud stage (day)	Bud stage in color (day)	Flower anthesis (day)	Flower longevity (day)	Flower bud number	Bud length (mm)	Bud diameter (mm)	Leaf number
I	Anm1, Anm3, Anm5, Anm7, Anm8, Anm9, Anm10, Anm12, Anm13, Anm14, Anm15, Anm17, Anm18	Group mean	16.95	64.41	11.77	8.10	7.43	1.00	10.96	7.30	9.35
		Z-score*	0.01	− 0.06	0.25	0.23	0.12	0.00	0.25	0.12	0.28
II	Anm2, Anm4	Group mean	13.33	70.00	10.83	6.00	8.67	1.00	10.06	8.43	6.67
		Z-score	− 0.45	0.23	− 0.23	− 0.80	0.84	0.00	− 0.01	0.64	− 0.93
III	Anm11	Group mean	19.00	85.67	10.67	8.00	8.33	1.33	10.81	8.26	10.33
		Z-score	0.27	1.05	− 0.31	0.18	0.65	2.95	0.21	0.57	0.72
IV	Anm6, Anm16	Group mean	19.00	58.33	8.83	6.00	3.83	0.83	4.10	3.36	6.00
		Z-score	0.27	− 0.37	− 1.27	− 0.80	− 1.98	− 1.45	− 1.75	− 1.72	− 1.23
Total mean			16.89	65.54	11.28	7.63	7.22	1.00	10.09	7.04	8.73

Cluster	Accession code		Leaf width (mm)	Leaf length (mm)	Petal number	Flower length (mm)	Flower diameter (mm)	Stem height (mm)	Stem diameter (mm)	Root length (mm)	Tuber fresh weight (g)
I	Anm1, Anm3, Anm5, Anm7, Anm8, Anm9, Anm10, Anm12, Anm13, Anm14, Anm15, Anm17, Anm18	Group mean	25.94	25.11	5.35	33.40	19.11	108.88	2.33	103.70	3.82
		Z-score*	0.03	0.02	0.37	0.17	0.35	0.23	0.37	0.02	− 0.14
II	Anm2, Anm4	Group mean	34.74	24.91	5.00	36.01	16.81	120.59	2.29	93.36	8.43
		Z-score	0.88	0.21	− 0.12	0.48	0.10	0.71	0.269	− 0.28	2.45
III	Anm11	Group mean	14.10	11.90	5.00	34.77	12.00	66.68	1.83	59.51	2.55
		Z-score	− 1.10	− 1.87	− 0.12	0.33	− 1.05	− 1.49	− 0.73	− 1.28	− 0.86
IV	Anm6, Anm16	Group mean	19.72	18.09	3.50	17.45	9.01	66.5	1.11	129.85	2.19
		Z-score	− 0.56	− 0.88	− 2.21	− 1.74	− 1.64	− 1.49	− 2.29	− 1.06	− 1.06
Total mean			25.57	23.58	5.09	31.99	17.34	103.14	2.17	103.00	4.08

*: Z-score was calculated as the difference between the mean of the trait (for each group) and the total mean, divided by the total standard deviation (SD). The highest positive Z-scores are associated with the relative highest value of the variable among the groups. Since the decrease in the value of phenological traits (except for flower longevity on plant) is a desirable feature, the groups with further negative Z-scores were considered to be more superior. Conversely, since the increase in the value of morphological traits is a desirable feature, the groups with further positive Z-scores were considered to be superior.

### Comparison of superior accessions under different pre-treatment conditions

The overall comparison of cluster analysis results under different pre-treatment conditions (non-chilling, chilling, GA_3_, and 5-azaC) showed that in all the four conditions, Anm3, Anm12, and Anm18 were relatively superior regarding most evaluated traits (Fig. 3). The results revealed that Anm3, Anm5, Anm12, Anm14, and Anm18 exhibited relatively higher values ​​compared with the total mean under chilling, GA_3_, and 5-azaC. In addition, Anm1, Anm3, Anm5, Anm8, Anm9, Anm10, Anm12, Anm13, Anm14, Anm15, and Anm18 had ​​higher values than the total mean in both GA_3_ and 5-azaC pre-treatment. Anm7 was the single accession with a value higher than the total mean under 5-azaC application conditions (Fig. 3).

#### Correlation coefficient between the studied indices of accessions under different pre-treatment conditions

To determine the best phenological and morphological indices for identifying the accessions with high ornamental values, the correlation coefficients between the traits were calculated under different pre-treatment conditions (Table 8). According to the results, the longevity of flower on the plant, as an important ornamental index, had a significant positive correlation with flower length under four pre-treatment conditions, namely non-chilling (r = 0.89), chilling (r = 0.57), GA_3_ (r = 0.62), and 5-azaC (r = 0.51). In addition, stem length and stem diameter showed a significant positive association with flower longevity under non-chilling, GA_3_, and 5-azaC conditions (Table 8).

**Table 8:** Correlation coefficient between flower longevity and morphological indices of *Anemone* accessions under different pre-treatment conditions.

Pre-treatment		Trait		
		Leaf length	Stem diameter	Stem height
Non-chilling	Flower longevity	0.89**	0.93**	0.91**
Chilling		0.57*	0.39	0.33
GA_3_(150 mg L^-1^)		0.62*	0.67**	0.69**
5-azaC (40 µM)		0.51*	0.55*	0.66**

*(Significant at 5% probability level) and ** (Significant at 1% probability level).

## Discussion

Iran is a country rich in germplasm resources of the genus *Anemone*^1^, known in floriculture as an early flowering geophyte. This genus has a high ornamental potential and can be utilized for commercial production. Therefore, development techniques for sustainable uses, such as propagation, forcing, and breeding, could play pivotal roles in conserving the genetic resources of *Anemone*. There are no reports of wild *Anemone* variation in Iran, and the present study is the first investigation focusing on flowering behavior of *Anemone* accessions in response to different pre-treatments in the world. The represented information herein could be employed by researchers, ornamental plant producers, and landscape designers to improve conservation strategies. Initiating breeding programs for commercial production requires understanding the physiological behavior of *Anemone* in the dormancy and flowering process of tubers.

The present data provided evidence for the flowering behavior among the studied wild accessions. Pre-treatment of tubers with GA_3_ reduced the sprouting time about 17 days earlier than that in non-chilling and chilling conditions. However, the fastest sprouting time (16.89 ± 7.83 days) was recorded for the treated tubers under 5-azaC. Using 5-azaC treatment, flower buds were visible about 65.54 ± 19.12 days after sprouting, followed by GA_3_ (75.02 ± 19.35 days), chilling (76.81 ± 21.72 days); the longest time (79.62 ± 45.56 days) until flower bud formation was detected in non-chilling treatment (Table 2). These findings revealed that chilling is not necessary for sprouting and flowering of most *Anemone* accessions; thus, regrowth and flowering occurred even in chilling-free condition (albeit with delay). Notwithstanding, there were no differences in sprouting time between chilling and non-chilling conditions, and hormonal pre-treatments significantly accelerated the sprouting process. For note, in our study, 5-azaC accelerated the bud formation whereas chilling and GA_3_ pre-treatment had insignificant effects (about 4 to 5 days) on bud formation. It has been confirmed that 5-azaC contributes to earlier flowering by reducing DNA methylation^32,33^. Overall, concerning the studied phenological traits of *Anemone* accessions, 5-azaC treatment more significantly reduced the vegetative growth period in comparison with other treatments, which is explained by the comparison of their growth period. Pre-treatment with 5-azaC lasted about 101 days from planting to flowering. Subsequently, GA_3_ pre-treatment required approximately 112 days to flowering. For chilling and non-chilling, the required time from planting to flowering was about 130 days from planting. Therefore, the one-month reduction in the growth period clearly indicated the positive effect of 5-azaC (Table 2). Based on the results, the lack of chilling increased the vegetative growth period of the plants. Moreover, the root was longer under the non-chilling condition. These results are in agreement with a previous study on three cabbage cultivars^41^ where 5-azaC decreased the germination and flowering time of cabbages.

Genes associated with vernalization and flowering are activated by DNA demethylation^35^. Transfer of plants from vegetative to flowering is an important process controlled by a network of genes in plants^42^. Vernalization in the plant could be effectively replaced by 5-azaC treatment and act as a flowering accelerator^43^. The 5-azaC either increases the amount of carbohydrate (leaf sugar content) through regulating the expression of carbohydrate metabolism genes and flowering genes^44^ or stimulates flowering by reducing inhibitor hormones, IAA for instance^45^. As mentioned in the results, 40 μM 5-azaC had a negative effect on the phenotype of *Anemone* accessions, reducing the plant height, root length, and the number of leaves and flower buds. A similar result was reported by Li et al.^35^ in regard to spinach. They reported that the presence of 5-azaC decreased the plant height and root length and accelerated flowering. Consistent with the present results, 5-azaC reduced the leaf number and length in *Erodium cicutarium*^46^ and resulted in early flowering in *Pharbitis nil*^32^. Conversely, 5-azaC treatment had a positive effect on flower diameter, stem diameter, and tuber weight. Our findings did not reveal any morphological abnormalities except for the decrease in height. Reduced DNA methylation could lead to abnormal plant phenotypes^47^. Our results are in line with those of Yingduan et al.^48^ who reported that phenotypic changes, such as dwarfing and small leaves, were triggered with 5-azaC treatment in wheat. Unlike our results, deformation of phenotype in *Jatropha curcas*^31^ and *Chrysanthemum*^33^ were reported.

Based on our results, GA_3_ (150 mgL^-1^) significantly increased the plant height, leaf number, petal number, and flower longevity in *Anemone* accessions (Tables 2, 3). It also reduced the sprouting time and vegetative growth period. External GA_3_ can act as a stimulator for internal GA_3_ synthesis^17^. Gibberellins play a role in breaking the dormancy, accelerating the germination and flowering time^49^ and increasing the vegetative growth by affecting the synthesis and activity of hydrolyzing enzymes in storage resources^50^. In addition, gibberellins improve the photosynthetic efficiency by enhancing photosynthetic enzymes and leaf area index and increasing the nutrient uptake^51^. Ferrante et al.^52^ reported that gibberellins were effective on reducing the breakdown of ribonucleic acid and protein, delaying aging, and increasing plant longevity via declining the production of ethylene. Hence, these reasons could justify the increase in the growth parameters of *Anemone* accessions under GA_3_ pre-treatment.

Accessions clustering under four pre-treatment conditions of tubers revealed a significant variation in the flowering behavior of *Anemone* accessions in response to non-chilling, chilling, GA_3_, or 5-azaC. Therefore, each area has its own management practices with regards to *Anemone*. The clustering of accessions from the same province or an adjacent origin into different clusters suggests the diversity of the assessed accessions. Our findings depicted that cluster analysis allowed the selection of superior accessions in all the pre-treatment conditions. The superiority of accessions Anm3, Anm9, Anm10, Anm11, Anm12, Anm13, Anm15, and Anm18 in non-chilling conditions (Table 4) indicated that these accessions could be utilized for cultivation in areas with short winters. It is noteworthy that most of the accessions superior in non-chilling conditions belonged to Abdanan region which has a subtropical climate and is located within the Zagros Mountains in the south of Ilam province, Iran. The excellent accessions in chilling conditions were Anm3, Anm5, Anm12, Anm14, Anm17, and Anm18 (Table 5), often distributed in areas with cold winters, for instance, Nahavand, Kerrend, and Ilam in Iran. It is expected that the use of these accessions in areas with long and cold winters result in optimal flowering. Ultimately, Anm3, Anm12, and Anm18 were selected as superior accessions in all the four pre-treatment conditions, which could be used as an important genetic resource in breeding programs. The distribution pattern (Kermanshah and Ilam) of these three accessions implied that they require low temperatures in winter for optimal growth and flowering. In cold periods, the above-mentioned accessions are also capable of growing without any restriction even in areas with short winters. Furthermore, these results shed light on the fact that a 90-day chilling was not sufficient for dormancy release in accession Anm2 in which sprouting was not observed in the differential response at this stage (belonging to *A. biflora* species). Tuber non-sprouting of this accession under non-chilling and chilling conditions indicated that further stimulus, such as longer periods of low temperature, is required for sprouting and flowering, which is confirmed by the stimulation of its growth and flowering under GA_3_ and 5-azaC. This accession was collected from Razavi Khorasan located in northeastern Iran. Higher latitude, cold, dry, and relatively long winters, and the overnight cold air until mid-spring, are the prominent features of Khorasan Razavi. These climatic conditions confirm that this species (*A. biflora*) of *Anemone* requires a longer period of cold to activate the growth stimuli. Exposure to GA_3_ and 5-azaC was able to meet this requirement.

Flower longevity on the plant is one of the important factors for the evaluation of ornamental plants. Understanding the relationships between the traits and identifying important and effective ornamental attributes help researchers select the superior accession. Generally, stem length and stem diameter were positively correlated with flower longevity on plants across non-chilling, GA_3_, and 5-azaC conditions. These results showed that these three indices could be considered as the criteria for selecting *Anemone* accessions with higher ornamental values. Thus, a tall accession with a thicker stem and a larger flower could be introduced as a superior accession.

## Conclusion

In conclusion, our findings introduced a novel and practical approach to accelerating flowering in off-season *Anemone* production. Based on the results, 5-azaC had an acceptable potential for reducing the growth period of *Anemone*. However, GA_3_ had a better effect on the improvement of the plant appearance. In the present study, most of the accessions did not require low temperatures for sprouting, and the flowering process was even performed without chilling; however, certain accessions for sprouting required vernalization and their flowering was found to be associated with low temperatures. The superior accessions were identified in terms of phenological and ornamental values in each pre-treatment condition. In total, Anm3, Anm12, and Anm18 were selected as superior accessions in all the four pretreatment conditions. Furthermore, this research could be a starting point for the design of novel strategies in order to develop effective conservation and management measures for a sustainable improvement in *Anemone*. Therefore, further studies are required to understand the behavior and mechanism of dormancy and flowering in *Anemone*.

## Material and methods

### Plant materials, experimental design, and cultivation process

All methods were performed in accordance with the relevant guidelines and legislation. From February to May 2018, intact tubers of 18 accessions related to *Anemone* during their flowering time were collected from six provinces of Iran (Table 9; Fig. 4). Generally, most of the Iranian accessions are geographically originated in Ilam province (12 accessions), followed by Lorestan (two accessions), Kermanshah (one accession), Khuzestan (one accession), Hamedan (one accession), and Razavi Khorasan provinces (one accession). In order to obtain representative samples and avoid collecting the clones of the selected accessions, an appropriate distance (200 m) was considered between the accessions in each site. The details of *Anemone* accessions are presented in Table 9. The identification of *Anemone* species was performed based on Flora Iranica^1^ and Colorful Flora of Iran^53^. *Anemone* tubers of each site were dried and stored in paper bags until the onset of the experiment. Tubers of these accessions were planted and grown in the experimental greenhouse of Ilam University (Ilam, Iran) with uniform conditions. The geographical coordinates of the location are 33.65°N latitude, 46.37° E longitude, and 2068 m altitude. The planting was carried out at the beginning of October 2018 utilizing tubers. The tubers of all the accessions listed in Table 9 are currently available to other researchers in the research greenhouse of Ilam University.

**Table 9:** Collection information for the 18 *Anemone* accessions studied.

No	Accession abbreviation	Province	Location site	Longitude (E)	Latitude (N)	Altitude (m)	Identified species
1	Anm1	Ilam	Abdanan-Sarabbagh	47° 33′ 56/014″	32° 53′ 55/556″	807	*A. coronaria*
2	Anm2	Razavi Khorasan	Mashhad- Ferizi	58° 58′ 18/183″	36° 29′ 04/354″	1673	*A. biflora*
3	Anm3	Kermanshah	Kerend-e Gharb	46° 15′ 19/673″	34° 17′ 39/710″	1681	*A. coronaria*
4	Anm4	Ilam	Ilam-Sirvan	46° 37′ 29/011″	33° 39′ 14/205″	1122	*A. coronaria*
5	Anm5	Ilam	Ilam-Chovar	46° 12′ 36/121″	33° 43′ 18/364″	1003	*A. coronaria*
6	Anm6	Lorestan	Khorramabad	48° 22′ 00/801″	33° 29′ 07/444″	1275	*A. coronaria*
7	Anm7	Lorestan	Borujerd- Kuh-e Garin	48° 39′ 01/4″	33° 52′ 10/000″	1571	*A.biflora*
8	Anm8	Ilam	Abdanan	47° 22′ 03/935″	32° 01′ 01/510″	992	*A. coronaria*
9	Anm9	Ilam	Abdanan -Dinar Kouh	47° 21′ 11/159″	32° 57′ 11/703″	1162	*A. coronaria*
10	Anm10	Ilam	Abdanan—Murmuri	47° 42′ 50/553″	32° 44′ 51/483″	452	*A. coronaria*
11	Anm11	Ilam	Eyvan- Gav Savar	46° 16′ 15/778″	33° 47′ 49/753″	1453	*A. coronaria*
12	Anm12	Ilam	Abdanan—Posht Ghale	47° 25′ 49/361″	32° 58′ 18/349″	862	*A. coronaria*
13	Anm13	Ilam	Chardavol- Shabab	46° 38′ 19/081″	33° 45′ 00/604″	965	*A. coronaria*
14	Anm14	Ilam	Murmuri- Abtaf	47° 49′ 11/2″	32° 39′ 24/7″	800	*A. coronaria*
15	Anm15	Ilam	Loumar- Tang-e Sazbon	46° 51′ 07/863″	33° 34′ 31/994″	901	*A. coronaria*
16	Anm16	Khuzestan	Dezful- Shahyun	48° 34′ 13/467″	32° 39′ 31/694″	654	*A. coronaria*
17	Anm17	Hamedan	Nahavand	48° 20′ 35/082″	34° 13′ 30/378″	1420	*A. coronaria*
18	Anm18	Ilam	Ilam Dam	46° 23′ 45/132″	33° 40′ 04/030	1080	*A. coronaria*

The accessions were evaluated for morpho-phenological traits as a factorial experiment in a completely randomized design (CRD) with three replications. We performed the experiment in four different pre-treatment conditions (non-chilling, chilling, 5-azaC, and GA_3_). Primarily, for artificial chilling pre-treatment, the tubers were dry stored at 4 °C (in refrigerator) for 13 weeks in dark conditions. Afterwards, 24 h before the planting, the chilling treated tubers were transferred to room temperature to start the experiment and forcing. The tubers of chilling and non-chilling pre-treatments were simultaneously soaked (24 h) in distilled water. Regarding 5-azaC (Sigma-Aldrich; 40 µM) and GA_3_ (Merck; 150 mgL^-1^) pre-treatments, 24 h prior to the cultivation, the tubers were immersed in the above-mentioned concentrations. Following the immersion time, to remove any residual materials on the surface of the tubers, they were washed thoroughly in distilled water. Subsequently, the tubers were individually potted into a black plastic pots (14 cm × 14 cm) filled with a mixture of peat moss, perlite, and coco peat (2:1:1) as growing media and placed in a greenhouse. Forcing conditions in the growth greenhouse were 23 °C/17 °C day/night temperatures with a RH 75% until flowering time. The pots of each treatment were randomly placed in the research greenhouse. All the plants were individually monitored on a daily basis over the whole period of the experiment. Normal horticultural practices, such as fertilizer application, irrigation practices, and pesticide application were applied during the experiment. As soon as the substrate surface of the pots was dried, irrigation was carried out during the growing season according to the need of the pots. Fertilizer (NPK; 20–20-20) was applied (2 mgL^-1^) three times at one-month intervals along with irrigation water. Aphids are among the key pests of *Anemone*, particularly at the bud stage. To control aphids during the experiment, we performed foliar application of diazinon insecticide (1 mgL^-1^) through hand sprayers.

### Phenological and morphological analysis

Phenological and morphological traits were measured once the plants reached the flowering stage. We recorded the studied phenological parameters, including days to sprouting (number of days from tuber planting to sprouting), days to bud stage (number of days from sprouting to flower bud appearance), days to bud stage in color (from bud appearance until bud showing color), days to flower anthesis (from colored bud to fully opened flower), and flower longevity on plant (from anthesis to wilting of petals). Morphological traits, such as flower bud number, bud length (mm), bud diameter (mm), leaf number, leaf width (mm), leaf length (mm), petal number, flower length (mm), flower diameter (mm), stem height (mm), stem diameter (mm), root length (mm), and tuber fresh weight (gr), were also measured. Digital caliper (Guanglu, resolution: 0.01 mm) and ruler were employed to measure these traits.

### Statistical analysis

Using SAS^54^ software version 9.453, analysis of variance (ANOVA) was performed on the accessions and pre-treatments as factors. Since the evaluated morpho-phenological parameters have different units of measurement scale, it is possible to compare different traits only following standardization and converting them into Z-scores. In order to avoid the effects of scale differences, the mean of each character was standardized using Z-score. Standardization data (Eq. 1) allow the comparison of different traits regardless of unit.

1 $$Z\_score = \frac{X - \mu }{\sigma }$$

In this equation, x, μ and σ are the raw score, mean, and standard deviation, respectively^55^. Z-scores of four treatments for different traits were used to compare the mean of the treatments and their effects on traits. A Z-score indicated the number of standard deviations by which the value of the mean of the treatment group is above or below of the total mean. The highest positive Z-scores were associated with the relative largest value of the variable among the groups.

Cluster analysis was performed based on Ward's method using squared Euclidean distance and discriminate analysis executed to identify the cutting point using SPSS^56^. In addition, the correlation coefficients between the traits were calculated using Pearson’s correlation coefficients with SPSS^56^.

## Supplementary Information

Supplementary Figures.
