# Supplementary material for: Exogenous 5-azaCitidine accelerates flowering and external GA3 increases ornamental value in Iranian Anemone accessions
Source: Sci Rep. 2021 Apr 5;11:7478. doi: 10.1038/s41598-021-86940-6 (PMC8021551; doi:10.1038/s41598-021-86940-6)
Supplement: Supplementary file 1 — Supplementary Figures. [file 41598_2021_86940_MOESM1_ESM.docx]

**Exogenous 5-azaCitidine Accelerates Flowering and External GA3 Increases Ornamental Value in Iranian *Anemone* Accessions**

**Vahideh Yari ^1^, Zeynab Roein^2^* Atefeh Sabouri^3^***

^1^Graduated MSc Student, Department of Horticultural Sciences, Faculty of Agriculture, Ilam University. Ilam, Iran. Email: v.yari@ilam.ac.ir

^2^Assistant Professor, Department of Horticultural Sciences, Faculty of Agriculture, Ilam University. Ilam, Iran. Email: z.roein@ilam.ac.ir

^3^Associate Professor, Department of Agronomy and Plant Breeding, Faculty of Agricultural Sciences, University of Guilan, Rasht, Iran. Email: a.sabouri@guilan.ac.ir

**Supplementary Figures**


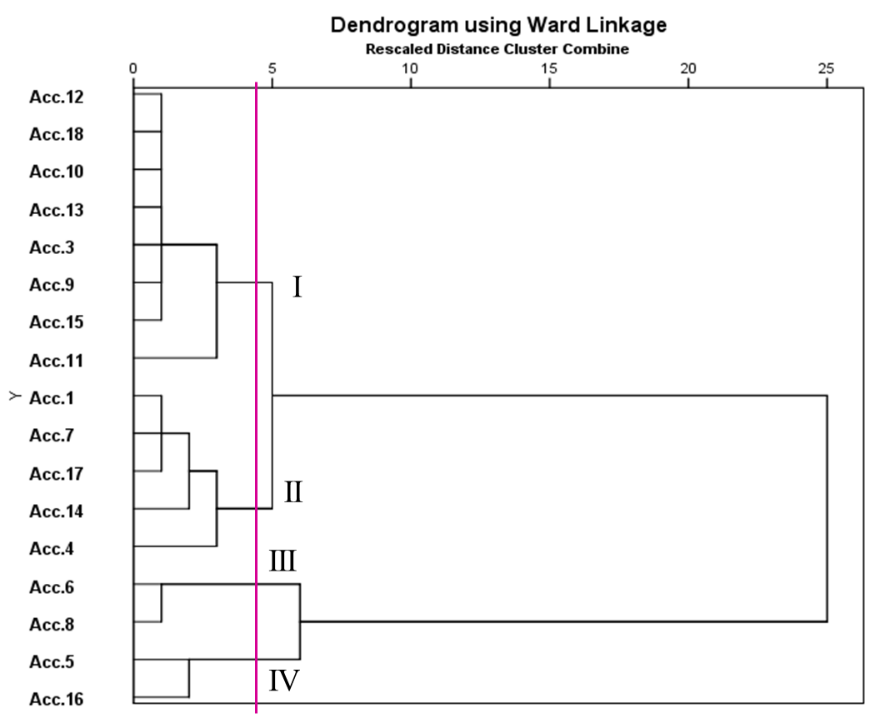


**Supplementary Figure 1.** Dendrogram of 18 *Anemone* accessions under non-chilling pre-treatment revealed by Ward cluster analysis


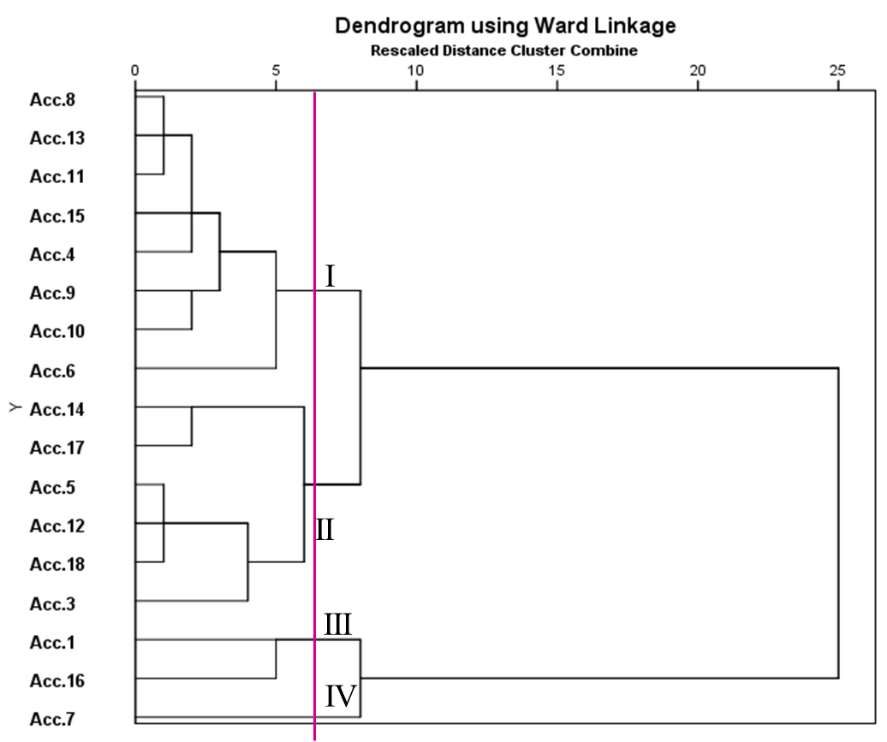


**Supplementary Figure 2.** Dendrogram of 18 *Anemone* accessions under chilling pre-treatment revealed by Ward cluster analysis


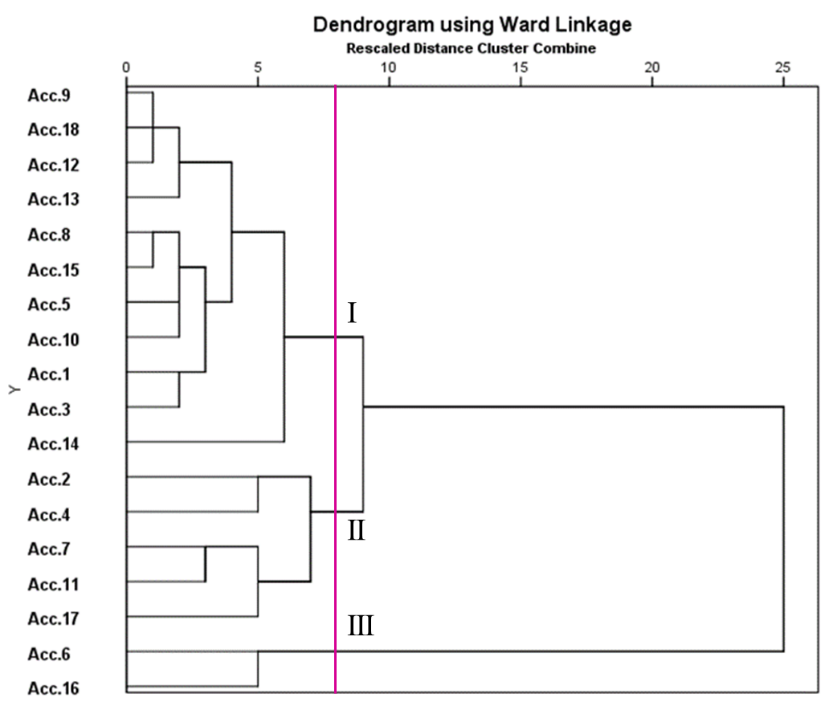


**Supplementary Figure 3.** Dendrogram of 18 *Anemone* accessions under GA_3_ pre-treatment revealed by Ward cluster analysis

**
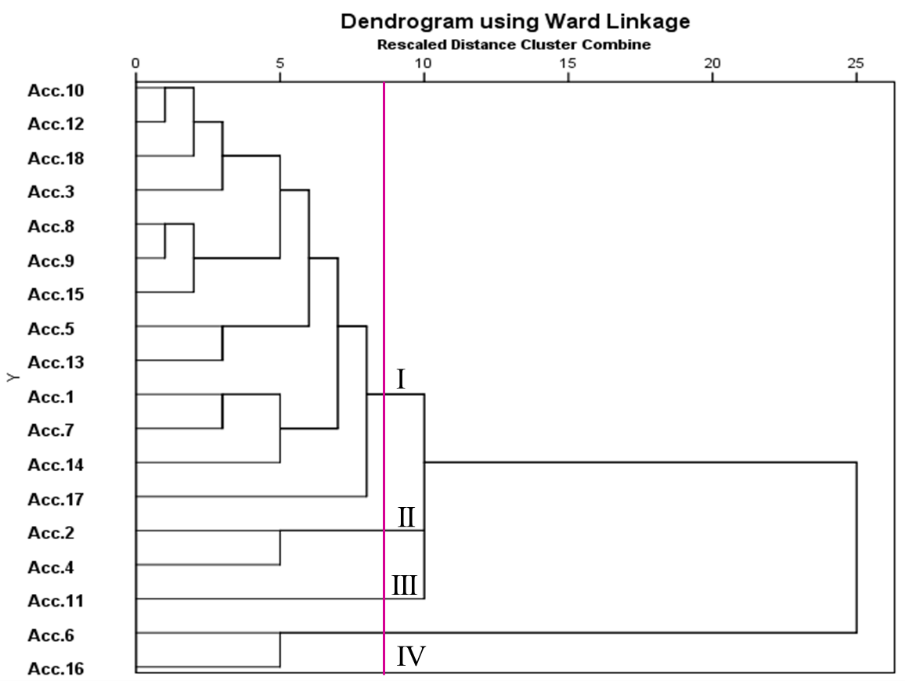
**

**Supplementary Figure 4.** Dendrogram of 18 *Anemone* accessions under 5-azaC pre-treatment revealed by Ward cluster analysis
